# Redox mechanism of glycerophospholipids and relevant targeted therapy in ferroptosis

**DOI:** 10.1038/s41420-025-02654-y

**Published:** 2025-08-01

**Authors:** Shuwei Chang, Minghui Zhang, Chang Liu, Mingyu Li, Yuefen Lou, Hexin Tan

**Affiliations:** 1 Department of Pharmacy, Shanghai Fourth People’s Hospital Affiliated to Tongji University, Shanghai, China; 2 Clinical Cancer Institute, Center for Translational Medicine, Naval Medical University, Shanghai, China; 3 Department Chinese Medicine Authentication, College of Pharmacy, Naval Medical University, Shanghai, China

**Keywords:** Cell death, Drug development

## Abstract

Ferroptosis, an iron-dependent form of regulated cell death driven by redox dysregulation, is defined by iron overload, reactive oxygen species overproduction, and subsequent peroxidation of polyunsaturated fatty acid-containing phospholipids, notably glycerophospholipids. This review comprehensively delineates the enzymatic such as lipoxygenases and non-enzymatic including Fenton reaction pathways governing glycerophospholipid peroxidation. Furthermore, we systematically dissect fine regulation of iron ions, including absorption, transport, and redox state transition. Given pathophysiological relevance of ferroptosis to numerous diseases, especially neurodegenerative disorders and various cancers, we evaluate emerging therapeutic strategies targeting key ferroptosis nodes, with a primary focus on the key enzymes involved in lipid peroxidation, transferrin receptor-mediated endocytosis mechanism and traditional Chinese medicine. Our work provides a direction for advancing ferroptosis research and developing combinatorial therapies that synergize ferroptosis induction with conventional treatments.

## Facts

- Ferroptosis, a distinct form of regulated cell death, is driven by iron overload and subsequent lipid peroxidation mediated by reactive oxygen species.
- Lipid peroxidation occurs through enzymatic and non-enzymatic pathways, generating free radicals and protein adducts etc. that directly drive ferroptosis.
- The metabolism regulation of glycerophospholipid containing polyunsaturated fatty acid chains critical for in ferroptosis.
- Iron ion homeostasis regulation is critically significant in ferroptosis.
- Ferroptosis is implicated in numerous diseases, such as neurodegenerative diseases, cardiovascular disorders, and cancers. Therapies targeting ferroptosis, particularly the transferrin receptor-mediated process, hold therapeutic promise. Traditional Chinese herbal medicine, with its multi-target bioactive compounds, has also emerged as a key modulator of ferroptosis.

## Outstanding Questions

- Ferroptosis, a multifactorial cell death mechanism linked to diverse pathologies, exhibits redox duality-either exacerbating or mitigating disease progression. Thus, how to control the balance between oxidation and reduction?
- Why do certain Traditional Chinese Medicine (TCM) active ingredients exert contradictory bidirectional effects on ferroptosis regulation? Reconciling their multi-target actions (e.g., simultaneous modulation of GPX4, ACSL4, Nrf2) to achieve precise ferroptosis control represents a critical challenge in TCM therapy.

## Introduction

Ferroptosis research originated in oncology, driven by 30% mutations in the RAS family of small GTPases in cancer cells [1]. Mechanistically, ferroptosis is initiated by redox activity of iron ions, which catalyze reactive oxygen species (ROXs) generation, leading to lipid peroxidation, this process involved in a variety of cellular processes, including: iron ions transport and transition of redox state, lipids redox homeostasis, metabolism activity and diet structure. In view of the complexity of ferroptosis and crosstalk with diverse signaling networds, ferroptosis dysregulation contributes to multiple pathologies: inflammation [2, 3], ischemic damage [4], cardiovascular diseases [5], kidney injury [6], degenerative pathologies [7, 8] and various cancer [9] (Table 1). Therefore, a systems understanding of its molecular drivers, from iron-lipid interplay to metabolic chaos, is essential for developing precision therapies that exploit ferroptosis modulation.

**Table 1:** Ferroptosis involves multiple diseases in different organs.

Organ	Disease type
	parkinson [203], alzheimer’s disease [204], epilepsy [205], neuroinflammation [206], dopaminergic neurons [207], blood-spinal cord barrier [208], neuropathic pain [209], brain injury [210], hemorrhagic stroke [211], subarachnoid hemorrhage [212], glioblastoma [213], etc.
	acute lung injury [214], asthma [215], tuberculosis [216], pneumonia [217], chronic obstructive pulmonary disease [218], pulmonary hypertension [219], non-small cell lung cancer [220], lung adenocarcinoma [221], lung squamous cell carcinoma [222], etc.
	heart failure [223], cardiomyopathy [224], cardiovascular disease [5], ischemia/reperfusion cardiac injury [225], coronary heart disease [226], septic heart injury [227], cardiac hypertrophy [228], etc.
	acute kidney injury [229], chronic kidney disease [230], diabetic kidney disease [231], kidney fibrosis [232], lupus nephritis [233], hypertensive nephropathy [234], cell renal cell carcinoma [235], kidney renal papillary cell carcinoma [236], etc.
	nonalcoholic fatty liver [237], liver fibrosis [238], liver injury [239], Metabolic dysfunction-associated fatty liver disease [240], acute liver failure [241], metabolic dysfunction-associated steatotic liver disease [242], liver cancer [243], etc.
	chronic atrophic gastritis [244], gastric ulcer [245], gastric mucosal injury [246], gastric cancer [247], etc.
	ulcerative colitis [248], intestinal barrier disfunction [249], intestinal ischemia-reperfusion injury [250], colonic inflammation [251], acute colitis [252], colorectal cancer [253], etc.
	acute pancreatitis [254], acute hypertriglyceridemic pancreatitis [255], pancreatic cancer [256], pancreatic ductal adenocarcinoma [257], etc.
	abnormal uterine bleeding [258], endometritis [259], early pregnancy loss [260], endometrial stromal cells inflammation [261], endometriotic cysts [262], recurrent spontaneous abortion [263], uterine corpus endometrial carcinoma [264], etc.

## lipid peroxidation in ferroptosis

### Molecular basis of lipid peroxidation

**Fig. 1:**
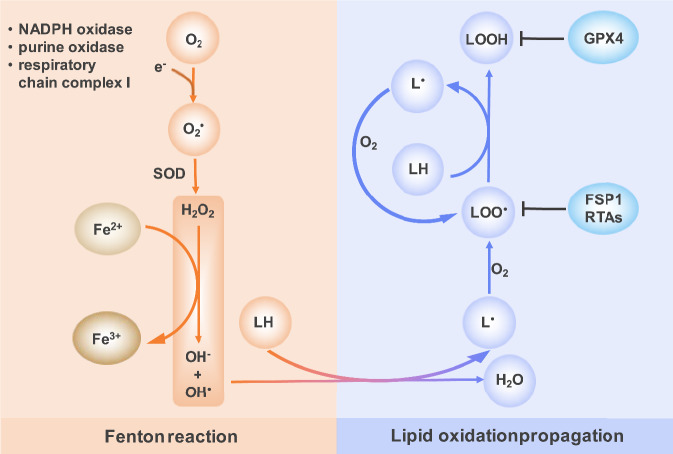
Mechanism of lipid peroxidation. Superoxide anion radicals are enzymatically produced by sources such as NADPH oxidase, purine oxidase, mitochondrial complex I etc. These radicals are then converted to hydrogen peroxide (H₂O₂) by superoxide dismutase (SOD). H₂O₂ reacts with Fe²⁺, oxidizing it to Fe³⁺ and producing the highly reactive hydroxyl radical (HO•) via the Fenton reaction. Hydroxyl radicals abstract hydrogen atoms from oxidizable molecules like polyunsaturated lipids (LH), yielding carbon-centered lipid radicals (L•). These rapidly react with oxygen (O₂) to form lipid peroxyl radicals (LOO•). LOO• propagate lipid oxidation by abstracting a hydrogen atom from adjacent polyunsaturated lipids (LH). This forms lipid hydroperoxides (LOOH) and generates a new lipid radical (L•), which rapidly reacts with oxygen to yield another peroxyl radical (LOO•), sustaining the chain reaction. Inhibiter of ferroptosis: GPX4, FSP1, RTAs. GPX4, glutathione peroxidase 4; FSP1, ferroptosis suppressor protein-1; RTAs, radical-trapping antioxidants.

Since its conceptualization in 2012, there has been an explosion of research surrounding ferroptosis [1]. As a distinct type of cell death, ferroptosis differs fundamentally from other types of cell death. Membrane perforins are characteristic features of other cell death modalities, such as BAX/BAK in apoptosis [10], MLKL in necroptosis [11], GSDM and NINJ1 in pyroptosis [12–15], however this is not the case for ferroptosis. As the name implies, ferroptosis is iron-dependent, and phospholipids that contain polyunsaturated fatty acids (PUFAs) are strongly driven by oxidation, leading to directly damage of cell membrane and ultimately cell death [16]. Superoxide anion is a product obtained by single-electron reduction of oxygen [17], and many proteins and enzymes are involved the process, such as, nicotinamide adenine dinucleotide phosphate (NADPH) oxidase [18], purine oxidase [19] and the respiratory chain complex I [20], etc. then superoxide anions can react with reducing substances or biological macromolecules, for example superoxide dismutase (SOD) to produce H_2_O_2_ [21]. With the ability to accept and donate electrons, iron was allowed to participate in the Fenton reaction, which generates the hydroxyl radical (HO•) that can indiscriminately attack biomolecules, including lipids (Fig. 1).

### Glycerophospholipids peroxidation in ferroptosis

**Fig. 2:**
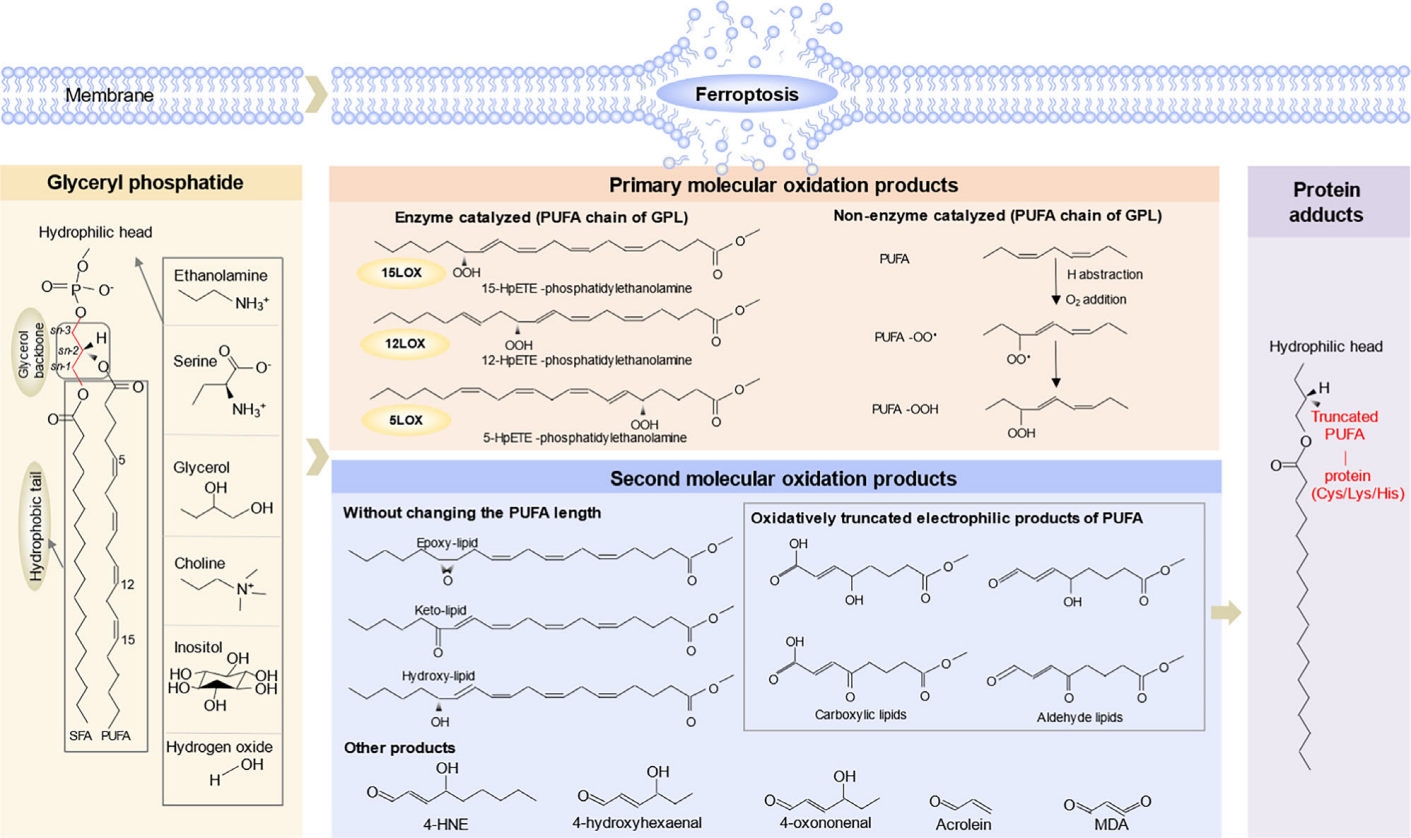
Peroxidation mechanisms of glycerophospholipids containing PUFA. Glycerol serves as the backbone of phospholipids, with two FA chains esterified at the sn-1 and sn-2 positions, and a polar head group attached at the sn-3 position. Common polar head groups in phospholipids include ethanolamine, serine, glycerol, choline, and inositol. PUFAs are typically esterified at the sn-2 position of phospholipids. Their structure, featuring two or more cis-configured double bonds, creates bis-allylic hydrogen atoms with low bond dissociation energies, making these sites highly susceptible to hydrogen abstraction. LP PUFAs undergo oxidation via both enzymatic (e.g., LOXs) and non-enzymatic ways, forming primary oxidized products. These primary products can be further redox to form the second products with intact FA chains (e.g., epoxy-, keto-, hydroxy-containing PLs). Or the electrophilic, chain-shortened products (e.g., carboxy, aldehyde relative PLs) due to the low dissociation energy of the O–O bond. Key byproducts of this process, notably 4-HNE and MDA (middle), readily form covalent adducts with nucleophilic residues on proteins (e.g., cysteine, histidine, and lysine), altering protein function and contributing to oxidative stress pathology (right). PUFA, polyunsaturated fatty acyl; GPL, glycerophospholipid; HpETE, 6 hloride 6 d 6 l-eicosatetraenoic acid; 4-HNE, 4-hydroxynonenal; MDA, malondialdehyde.

Phospholipids, key structural components of biological membranes, are primarily classified as glycerophospholipids (GPLs) or sphingomyelin [22]. Regarding GPLs, usually, there is a saturated fatty acyl (SFA), such as palmitic acid (C16:0), stearic acid (C18:0) at the sn-1 position of glycerol backbone, whereas there has a greater diversity at the sn-2 position, could be a SFA; a monounsaturated fatty acyl (MUFA), which has been shown to inhibit ferroptosis [16, 23]; or a polyunsaturated fatty acyl (PUFA), for example arachidonic acid (AA, C20:4), adrenic acid (AdA, C22:4) [24], eicosapentaenoic acids (EPA, C20:5), docosahexaenoic acid (DHA, C22:6), which contains multiple double bonds, are susceptible to peroxidation and particularly able to facilitate ferroptosis. GPLs are categorized by the hydrophilic head groups at the sn-3 position. GPL head groups-including phosphatidylcholine (PC), phosphatidylethanolamine (PE), phosphatidylserine (PS), phosphatidyl glycerol (PG), phosphatidylinositol (PI), and phosphatidic acid (PA) (Fig. 2), PC was mainly present in the extracellular leaflet, whereas PS and PE were exclusively in the cytoplasmic leaflet [25], given that PS acts as a “eat me” signal [26], once flipped into the outside of the phospholipid bilayer, it may indicate that the cell is in poor condition, such as apoptosis [27]. In addition, polyunsaturated glycerophospholipids (PUFA-GPLs) and specifically PUFA-PEs which have been identified as the selective targets of pro-ferroptotic lipid peroxidation [28].

Oxidation of PUFAs at the sn-2 position of GPLs proceeds via two distinct mechanisms: enzyme-dependent pathways (lipoxygenases, LOXs) and non-enzymatic dependent modes [29, 30]. For LOXs, are kinds of dioxygenases of iron-dependent, PUFAs are used as their substrates, and are named after the oxidized carbon arrangement number on the PUFA chain, such as 5LOX, 12LOX, 15LOX. Furthermore, functional arachidonate LOX genes have been identified within the human genome, underscoring their critical role in lipid peroxidation and ferroptosis [31]. The peroxidation susceptibility of PUFAs in GPLs is critically determined by their bis-allylic positions-carbon centers flanked by two double bonds, where the low bond dissociation energy facilitates hydrogen abstraction [32]. After oxidation, the main molecular product GPL-OOH is formed (Fig. 2). In mammalian cells AA (C20, 4) is one of the most abundant polyenoic fatty acids (FAs) that serve as substrates for the different mammalian LOXs [33], after oxidized by 15LOX, 12LOX and 5LOX, the HOO-group at the 15th, 12th or 5th carbon of the arachidonoyl residue generates 15/12/5-HpETE-phosphatidylethanolamine, respectively (Fig. 2), these products were detected to accumulate in cells undergoing ferroptosis [24, 34]. This suggests that enzyme-catalyzed lipid peroxidation plays an important role in ferroptosis, especially 15LOX [35, 36].

However, polyunsaturated PLs are inherently unstable due to the weak -O-O bond energy of GPL-OOH and readily undergo secondary free radical reactions, giving way to numerous truncated reactive products, including electrophilic oxidatively truncated lipids (epoxy/keto/hydroxy-lipids), or short leaving fragments without the signatures of parental phospholipid identity, such as 4-hydroxynonenal (4-HNE), 4-oxononenal, acrolein and malondialdehyde (MDA) etc [28]. And then, adduct formation occurs between electrophilic oxidatively truncated lipids and nucleophilic amino acid residues (e.g., histidine, lysine, and notably cysteine) in proteins [28]. These adducts confer increased hydrophobicity and high membrane affinity upon the modified proteins, thereby promoting ferroptosis [6].

## Glycerophospholipid (GPL) metabolism and regulation

### Phospholipases function in ferroptosis

**Fig. 3:**
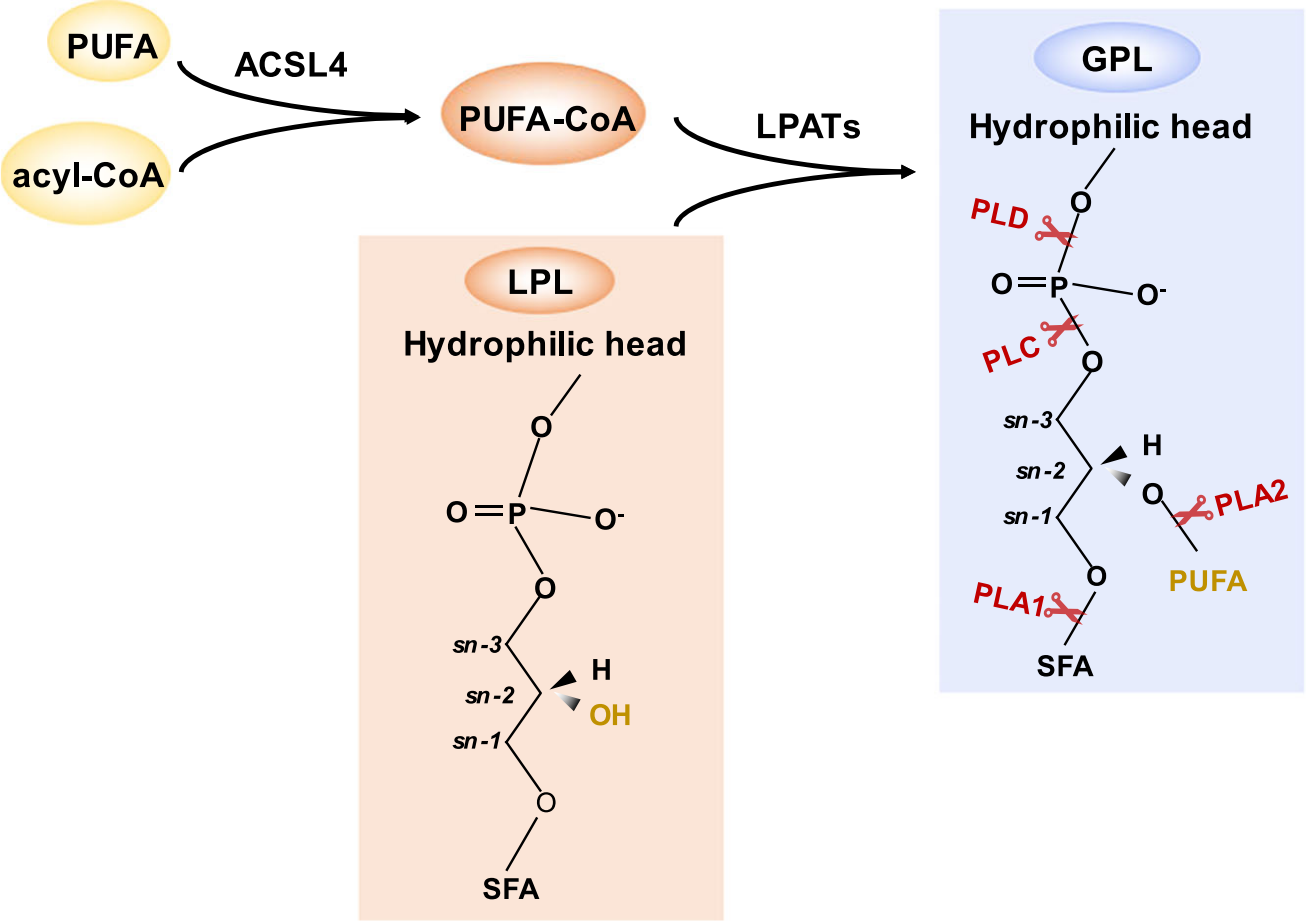
Glycerophospholipid (GPL) regulation. ACSL4 catalyzes PUFA and acyl-CoA to form PUFA-CoA, which then forms GPL with LPL in the presence of LPAAT enzyme. GPLs is hydrolyzed by phospholipases at specific sites: PLA1 cleaves the sn-1 fatty acyl ester, PLA2 the sn-2 fatty acyl ester, PLC the phosphodiester bond between glycerol and the phosphate group, and PLD the phosphodiester bond between phosphate and the head group. PUFA polyunsaturated fatty acid, ACSL4 acyl-CoA synthesis long-chain family member, LPL lysophospholipid, LPLATs lysophospholipid acyltransferases, GPL glycerophospholipid, SFA saturated fatty acid.

As peroxide substrates, it is of great significance to investigate the metabolism and regulation of GPLs in ferroptosis [37]. The core structure of GPL comprises three parts: backbone (glycerol), hydrophilic head (ethanolamine/serine/glycerol/choline/inositol/ hydrogen oxide) and two hydrophobic tail (Fig. 2), and they are catalyzed by phospholipase A, phospholipase C, and phospholipase D, respectively [22] (Fig. 3). Among the phospholipases, phospholipase A2 (PLA2) [38] critically regulates ferroptosis by hydrolyzing the sn-2 ester bond of glycerol. For example, Mihee et al proved that darapladib, an inhibitor of lipoprotein-associated phospholipase A2 (LpPLA2), synergistically induces ferroptosis in the presence of GPX4 inhibitors [39]; Ca²⁺-independent phospholipase A2β (IPLA2β) suppresses ferroptosis by hydrolyzing 15-HpETE-PE, while its genetic or pharmacological inactivation sensitizes cells to ferroptotsis [40]; By resisting ferroptosis, PLA2 family members regulate pathogenesis across multiple diseases: cancer [41, 42], brain injury [43], acute liver injury [44], neurodegenerative disease [45]. In addition, it was found that PLD, which is responsible for hydrolyzing the bond between hydrophilic head and phosphoric acid (Fig. 3), regulates ferroptosis signal transduction in mouse spleen hypoxia response [46].

### ACSL function in ferroptosis

The oxidation of GPL in the process of ferroptosis mainly focus on PUFA, so establishing GPL-PUFA metabolism is particularly critical. Generally, fatty acid (FA) synthesis is initiated by the condensation of malonyl-CoA with acetyl-CoA, followed by multiple elongation steps with two-carbon units to yield long chain FA, then desaturation and elongation of these FA chains result in different FA isomers (for example, ω-7 or ω-9 C18:1) [22, 47]. However, desaturases which generate ω-3 and ω-6 FAs are absent in mammals, therefore, the related precursors must be obtained from dietary.

Acyl-coenzyme A (acyl-CoA) synthases (ACS), which catalyze the conversion of FAs to fatty acyl-CoAs [48], are classified into very long-chain acyl-CoA synthase, acyl-CoA synthase long-chain family member (ACSL; for FAs with 10 to 22 carbons), medium-chain acyl-CoA synthase, and short-chain acyl-CoA synthase based on the carbon chain length of their substrates [49]. ACSL comprises five isoenzymes: ACSL1 and ACSL3-6 [50]. Among ACSL family members, ACSL4 specifically esterifies CoA to PUFAs, including arachidonic acid (AA; forming AA-CoA), adrenic acid (AdA), eicosapentaenoic acid (EPA), docosahexaenoic acid (DHA), epoxyeicosatrienoic acids (EETs), and hydroxyeicosatetraenoic acids (HETEs), to generate PUFA-CoAs [51]. These PUFA-CoAs are subsequently esterified into phospholipids (forming PUFA-PLs) by lysophospholipid acyltransferases [52], such as lysophosphatidylcholine acyltransferases (LPCAT) [53, 54] (Fig. 3), PUFA-PLs are readily to be oxidized and trigger ferroptosis [55, 56]. Cheng et al. demonstrated that ACSL4 overexpression elevated levels of ferroptotic markers, including 5‑ hydroxyeicosatetraenoic (HETE), 12‑HETE, and 15‑HETE, and thereby suppressed glioma cell proliferation [57]. Furthermore, accumulating evidence links ACSL4 to the pathogenesis of multiple diseases via ferroptosis [58–61]. Beyond ACSL4, other family members also modulate ferroptosis. For example: ACSL1 promotes α-eleostearic acid (Αesa) incorporation into neutral lipids (e.g., triacylglycerols); disrupting triacylglycerol biosynthesis suppresses Αesa-induced ferroptosis [62]; Inhibiting ACSL3 reduces ferroptosis susceptibility in clear cell renal cell carcinoma (ccRCC) cells [63].

### LPLAT function in ferroptosis

The chemical diversity of phospholipids stems primarily from variations in headgroups at the sn-3 position and FAs at the sn-1 and sn-2 positions of glycerol-based structures. Glycerol-3-phosphate acyltransferases (GPATs) and lysophospholipid acyltransferases (LPLATs) (Fig. 3), working in concert with phospholipase enzymes, contribute to this diversity via selective esterification and deesterification [64]. To date, 14 LPLATs have been identified, all fall into one of two enzyme families: the 1-acylglycerol-3-phosphate O-acyltransferases (AGPATs) or the membrane-bound O-acyltransferases (MBOATs) [65]. Given their role in phospholipid remodeling, LPLATs are closely linked to ferroptosis. Specifically, LPCAT3 (MBOAT5) is responsible for generation of C20:4 phospholipids. Inhibiting LPCAT3 induces rapid remodeling of polyunsaturated phospholipids in human cells, characterized by suppressed C20:4-PLs and a compensatory increase in C22:4-PLs. This lipid rewiring provides a mechanistic basis for the observed partial, yet incomplete, protection from ferroptosis [66]. LPCAT3 is also involved in subarachnoid hemorrhage induced early brain injury and associated with ferroptosis through suppress and upregulate LPCAT3 expression level [67]. Moreover, LPCAT3 is significantly upregulated in various cancers and emerges as a potential prognostic biomarker in acute myeloid leukemia, it may be correlated with immune infiltration and ferroptosis [68]. For LPCAT2, its overexpression enhances ferroptosis susceptibility in HEK293T cells [53]. However, LPLATs incorporate not only PUFAs but also SFAs into GPLs. For example, Hyemin et al. reported that LPCAT1 specifically utilizes SFA-CoA to acylate lysophospholipids, generating SFA-phospholipids that confer ferroptosis resistance by displacing ferroptosis-susceptible PUFAs [69]; similarly, Ziwen Li et al. demonstrated that LPCAT1 confers ferroptosis resistance by enhancing membrane phospholipid saturation via the Lands cycle, thereby reducing PUFA incorporation [70].

## Iron transporters and related regulation

### Iron absorption, circulation and storage

Iron, a biometal with pleiotropic functions, is central to ferroptosis execution while simultaneously mediating oxygen transport (via hemoglobin), supporting erythropoiesis, and participating in mitochondrial electron transport chains [71], synthesis of iron-sulfur proteins, mitochondrial function, DNA synthesis, etc [72]. In humans, >90% of irons demand is met by recycling existing iron, cause of the total iron flux required to maintain erythropoiesis is about 2–3 × 10^15^ atoms of iron per second in an adult human [73]. Given the inevitable physiological iron loss, such as perspiration, epithelial desquamation, menstruation, stringent regulation of iron metabolism, including absorption (via DMT1), transport (transferrin-mediated), redox cycling, storage (ferritin-bound), and systemic homeostasis is essential to prevent deficiency [74].

**Fig. 4:**
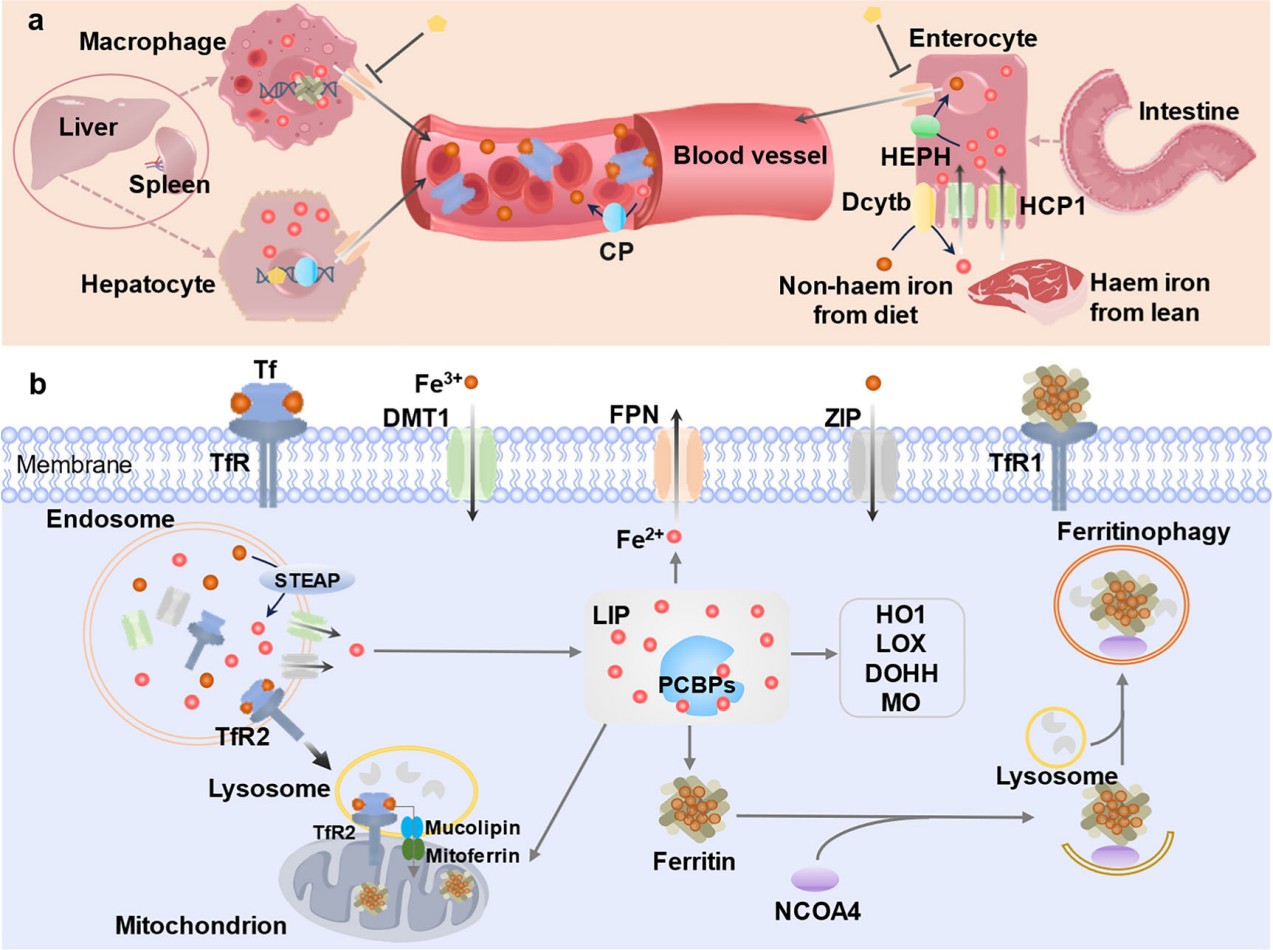
Irons homeostasis regulation. **a** Systemic iron flux. Macrophages in the liver and spleen take up and digest senescent red blood cells, releasing the iron into the plasma; the remainder comes from dietary sources. Dietary iron comprises heme iron (from lean) and non-heme iron. Intestinal enterocytes absorb heme iron via HCP1, while non-heme iron is absorbed by DMT1 after being reduced to Fe²⁺ by Dcytb, which is primarily localized in the enterocyte’s apical membrane. FPN, the only known iron ions exporter, which mediates Fe^2+^ export in mammalian cells. Multicopper oxidases oxidize Fe²⁺ to Fe³⁺. HEPH (highly expressed in intestines) and CP (hepatocyte-derived, circulating in plasma) both exhibit ferroxidase activity. HEPH presumably works in conjunction with FPN. **b** Cellular iron regulation. Tf binds Fe³⁺ and delivers it to cells via TfR-mediated endocytosis, forming endosomes. Within endosomes, Fe³⁺ dissociates from Tf, is reduced to Fe²⁺ by STEAP3, and is transported into the cytosol by DMT1 or ZIP transporters. Human TfR1 was found belonging to cell surface receptor for H ferritin. TfR2 contains mitochondrial targeting motif, facilitates irons delivery to the mitochondria; Lysosomes containing TfR2-α translocate to mitochondria and dock with them, facilitating iron transfer. Cytosolic labile iron is stored within ferritin cages with the assistance of PCBPs. During iron deficiency, ferritin interacts with the receptor NCOA4 and is degraded via ferritinophagy. Furthermore, PCBPs assist in other cellular iron processes: transferring iron from the importer DMT1, exporting iron via FPN, degrading heme through HO1; and regulating iron-dependent enzymes involved in lipid peroxidation (LOXs); other enzymes including DOHH, MO or enzymes in mitochondria. Tf transferrin, TfR transferrin receptor, FPN ferroportin, DMT1 divalent metal transporter 1, Dcytb duodenal cytochrome b, ZIP ZRT/IRT-like protein, STEAP six-transmembrane epithelial antigen of the prostate. HCP haem carrier protein 1, CP Ceruloplasmin, HEPH Hephaestin, LIP Labile ion pool, PCBPs (poly(Rc)-binding proteins 1-4), HO1 heme degradation via heme oxygenase 1, LOXs lipoxygenases, DOHH deoxyhypusine hydroxylase, MO monooxygenases.

Hepatic and splenic macrophages phagocytosing senescent erythrocytes, extracting iron from heme, and exporting it to the plasma; the remaining systemic iron pool is predominantly replenished from diet [73] (Fig. 4a). Dietary iron is categorized into heme and non-heme forms. Intestinal enterocytes absorb heme iron directly via haem carrier protein 1 (HCP1), while non-heme iron is transported by divalent metal transporter 1 (DMT1) after were reduced by reductase such as duodenal cytochrome b (Dcytb) to ferrous ions (Fe^2+^) (Fig. 4a); Dcytb, primarily located in enterocyte apical membranes, functions as a ferric reductase that converts ferric ions (Fe³⁺) to absorbable Fe²⁺ [75]. Ferroportin, the sole iron exporter in mammals, transports Fe²⁺ from cells and is critical for systemic iron homeostasis [76]. Multicopper oxidases, including intestinal-enriched hephaestin (HEPH), liver-derived plasma ceruloplasmin (CP), and placental-enriched Zyklopen, oxidize Fe²⁺ to Fe³⁺ through ferroxidase activity [77, 78]; hephaestin presumably works in conjunction with ferroportin [79].

Transferrin (Tf) facilitates cellular iron uptake by binding Fe³⁺ and undergoing Tf receptor (TfR)-mediated endocytosis, forming iron-loaded endosomes for furture intracellular transport [80–82] (Fig. 4b). Fe^3+^ dissociates from Tf and is reduced to Fe^2+^ by metalloreductases six-transmembrane epithelial antigen of the prostate 3 (STEAP3). The Fe^2+^ are subsequently transported into the cytosol via DMT1 or Zrt- and Irt-like protein (ZIP) transporters [72, 83, 84]. In addition, TfR2 facilitates irons delivery to the mitochondria, which a variety of enzymes are located there, such as, NADH-coenzyme Q oxidoreductase (complex I) and succinate-Q oxidoreductase (complex II), require iron as an essential cofactor; additionally, mitochondria consume iron for heme biosynthesis and iron-sulfur cluster assembly; mitochondrial targeting motif exists in the intracellular domain of TfR2-α, so the lysosomes containing TfR2-α move toward and physically interact with mitochondria to facilitate iron transfer [85–87]. The cytosolic labile iron pool (LIP) is regulated through dual mechanisms: under iron-replete conditions, excess labile iron is sequestered within ferritin nanocages via poly(Rc)-binding proteins 1-4 (PCBPs) mediated packaging [88–91]; conversely, during iron deficiency, NCOA4 acts as a autophagy receptor that directs ferritin to degradation by ferritinophagy [92] (Fig. 4b). PCBPs function as multifunctional chaperones coordinating distinct iron regulatory pathways: first, iron homeostasis: mediate ferritin-dependent storage, DMT1-mediated cellular iron import, and ferroportin-driven export; second, metabolic regulation: facilitate heme catabolism via heme oxygenase 1 (HO1) and modulate lipid peroxidation through LOXs; third, enzymatic coordination: support iron-dependent enzymes including deoxyhypusine hydroxylase (DOHH), monooxygenases (MO), and other enzymes in mitochondria [6, 90] (Fig. 4b).

### Transferrin (Tf) and Transferrin Receptors (TfRs)

Tf, a glycoprotein critical for systemic iron transport, comprises three subtypes: serum Tf, lactotransferrin, and melanotransferrin [93], this review focuses on serum Tf. Iron-loaded Tf binds to TfRs which locate on the cell surface, facilitating receptor-mediated endocytosis. This process enables the delivery of Fe³⁺ from the bloodstream into tissues such as the liver, spleen, and bone marrow [74]. Structurally, Tf consists of two lobes [94], and each binds a single Fe³⁺ ion, resulting in three possible iron occupancy states: binding one Fe^3+^, binding two Fe^3+^ (holo-Tf), and binding none Fe^3+^ (apo-Tf) [85]. In addition, the interaction between Fe³⁺ and Tf is pH-dependent: Fe^3+^ efficiently binds to Tf at Ph 7.4 and dissociates at acidic Ph in endosomes [95, 96]. Mammals express two Tfs, TfR1 and TfR2 [85]: TfR1 is ubiquitously expressed and especially highly in erythroblasts [71], given that over 80% of plasma Tf-bound iron is absorbed by erythroid precursors in the bone marrow, where it is utilized for heme synthesis [97]; however, TfR2 remained unidentified until in 1999 [98], the human TfR2 gene is expressed selectively in hepatocytes and erythroid [99].

Given the critical physiological role of Tf and TfRs in iron transport, their dysregulation has been implicated in the pathogenesis of various diseases, including anemia [100], renal fibrosis [101], systemic lupus erythematosus [102], hemochromatosis [103], among others. Such associations underscore the critical importance of tightly regulating Tf and TfRs, not only to preserve iron balance but also to mitigate disease progression, highlighting their potential as therapeutic targets. TfR expression is regulated through the IRP (iron regulatory protein)/iron-response element (IRE) axis in response to cellular iron status [104]; Under low iron conditions, IRP stabilizes TfR1 mRNA by binding to IREs in the 3’ untranslated region (3’ UTR), enhancing TfR1 protein synthesis and boosting cellular iron uptake [105]. Research demonstrates HIF signaling pathway upregulates TfR expression [106–108]. Furthermore, miRNAs are also involved in the TfRs regulation. For instance, miR-148a levels negatively correlate with TfR1 mRNA in hepatocellular carcinoma [109]; Bioinformatic analysis reveals that miR-497-5p inversely correlates with TfR in cervical cancer [110].

### Ferroportin function and related regulation

As mentioned above, dietary iron absorbed by intestinal enterocytes and recycled iron from macrophages phagocytosing aged erythrocytes are transported to systemic circulation via ferroportin-mediated export, as detailed below [111].

Ferroportin (FPN/SLC40A1 [79]) the primary cellular iron exporter, is abundantly expressed in key iron-handling tissues: duodenal enterocytes (dietary absorption), hepatocytes (storage), splenic macrophages (erythrocyte recycling), and placental syncytiotrophoblasts (maternal-fetal transfer) [73]. FPN critically regulates systemic iron homeostasis, as evidenced by transgenic models: FPN knockout mice exhibit enterocyte/macrophage/hepatocyte iron overload and embryonic lethality [79]; Hepatic FPN specific knockout mice under iron deficiency develop impaired iron mobilization and severe anemia, with red blood cell and hemoglobin levels much lower, demonstrating FPN’s dual role in hepatocyte iron mobilization and macrophage iron recycling during dietary iron stress [112]. Beyond anemia and hemochromatosis [113], FPN dysregulation contributes to multisystem pathologies: its deficiency induces memory impairment by promoting ferroptosis in Alzheimer disease [114]; while hepatic FPN downregulation drives proliferation and M2-like polarization of macrophages and leading to hepatic fibrosis through increased the levels of the M2 markers CD206, TGF-β, VEGF, MMP-9, Laminin, Collagen, IL-4 and IL-10 [115], etc. So as the sole iron exporter, FPN is strictly regulated.

Hepcidin, a 25 amino acid hepatocyte-derived peptide hormone, critically regulates FPN according to iron concentration [116]. Elevated plasma iron concentration triggers hepcidin upregulation, which binds FPN to induce its internalization and degradation, reducing iron export from enterocytes and macrophages into circulation. Conversely, decreased serum iron suppresses hepcidin production, enhancing dietary iron absorption (enterocytes) and hepatic iron mobilization (hepatocytes) via FPN-mediated efflux into circulation [72, 117]. Structurally, FPN functions as an electroneutral H⁺/Fe²⁺ antiporter, coupling each Fe²⁺ export to counter-transport of two protons; perturbation of either ion binding site uncouples this co-transport [118]. Hepcidin binds FPN in an outward-open conformation and completely occludes the iron efflux pathway to inhibit transport; hepcidin binding to FPN is coupled to iron binding, with an 80-fold increase in hepcidin affinity; the carboxy terminus of hepcidin directly contacts the divalent metal in FPN’s C domain, establishing a degradation targeting mechanism exclusive to iron-loaded FPN [119]. Research confirmed that hepcidin binding triggers rapid FPN ubiquitination in both FPN-overexpressing cell lines and murine bone marrow derived macrophages [120].

In addition, research has found that human FPN binds to and mediates Ca^2+^ transport; Ca^2+^ binding site distinct from Fe^2+^ binding sites; Ca^2+^ transport is significantly inhibited in the presence of Fe^2+^ but not vice versa; this indicate function of FPN as a Ca^2+^ uniporter may allow regulation of iron homeostasis by Ca^2+^ [121, 122]. Vamifeport (VIT-2763), the first clinical-stage oral FPN inhibitor, binds FPN’s central site, structurally overlapping hepcidin’s interaction site, competitively blocking hepcidin binding, this therapeutic is under clinical evaluation for β-thalassemia and sickle cell disease [123]. Namgaladze et al. demonstrated that Nrf2 (redox-sensitive transcription factor) accumulation coupled with BACH1 (transcription factor) downregulation induces FPN expression, conferring ferroptosis resistance in human macrophages [124] etc. Collectively, these findings validate FPN as a high-potential therapeutic target.

### Ferritin function and related regulation

Ferritin, as an iron ion chelating protein, sequesters iron ions to prevent lipid peroxidation caused by Fenton reaction, serving as a critical antioxidant safeguard in ferroptosis [125]. Ferritin, a ubiquitous protein found in all cells, forms multiple isoferritins composed of 24 subunits of two principal types: the H subunit (originally isolated from human heart and the predominant form there, also characterized by its heavier electrophoretic migration) and the L subunit (originally isolated from human liver and the predominant form there, with lighter electrophoretic migration) [126]. While the H subunit gene resides on chromosome 11q and the L subunit gene on chromosome 19q, the H:L ratio incorporated into the assembled ferritin complex varies dynamically depending on tissue type and developmental stage [88, 127].

Within the body’s iron transport system, FPN acts like export vehicles (trucks/ships), transporting irons out of absorption sites (enterocytes) and storage cells (like hepatocytes and macrophages) into the bloodstream. Conversely, the Tf/TfR system functions as import vehicles (delivery vans/trains), transporting irons from the blood into various cells throughout the body for utilization. Meanwhile, ferritin serves as the essential warehouse, storing and stabilizing irons within cells. Its remarkable capacity-each molecule can sequester up to 4500 iron atoms-makes it the central hub for safe, bioavailable iron storage [126]. Addition, ferritin also has ferroxidase activity, converting Fe^2+^ to Fe^3+^ with ferritin H chain but not L chain [88]. Ferritin is predominantly localized in the cytoplasm, but small quantities are in serum [128, 129], mitochondrial [130, 131] and nuclear [132, 133]. As macrophages phagocytose senescent red blood cells, releasing large quantities of irons into cytoplasm, and macrophages synthesize ferritin to sequester these irons. Additionally, macrophages secrete a portion of ferritin into the circulation [85, 134]. Beyond above, ferritin exhibits functional parallels with the Tf/ TfR system. Similar to Tf, serum ferritin can deliver iron to cells by binding to dedicated cell surface receptors. Known ferritin receptors include: first, TIM-2 (T cell immunoglobulin-domain and mucin-domain 2) specifically bound ferritin H [135]; second, Scara5 (a scavenger receptor that can bind various ligands) preferentially binds ferritin L [136], then enters cells through internalization; third, surprisingly, human TfR1 was identified as a cell surface receptor for H ferritin [137].

## Associated therapy

### Internal inhibition mechanism of ferroptosis

As an iron-dependent and non-apoptotic cell death, ferroptosis represents a promising therapeutic strategy. Modulation, either inhibiting or promoting its induction, holds potential efficacy, according to different diseases and therapeutic goals. The core pathway for ferroptosis inhibition centers on the System Xc⁻/GSH/GPX4 axis; System Xc⁻, an antiporter, imports extracellular cystine in exchange for intracellular glutamate. Cytosolic cystine is rapidly reduced to cysteine, the rate-limiting precursor for glutathione (GSH) synthesis. GSH acts as an essential cofactor for glutathione peroxidase 4 (GPX4), enabling GPX4 to catalyze the reduction of lipid hydroperoxides (LOOH) to corresponding alcohols (LOH), this process is critical for neutralizing membrane lipid peroxidation, the hallmark of ferroptosis [138, 139] (Fig. 1). Furthermore, researches have definitively established that selenium is required for GPX4’s catalytic activity [140, 141]. Critically, seminal research identified two key small-molecule compounds erastin and RSL3 (RAS-selective lethal compound 3) targeting System Xc⁻ SLC7A11 subunit and GPX4 respectively to induce ferroptosis [1, 139, 142]. The ferroptosis suppressor protein 1 (FSP1), operating within the FSP1-CoQ_10_-NAD(P)H axis, functions as an NAD(P)H-dependent oxidoreductase that catalyzes the reduction of membrane-bound ubiquinone (CoQ_10_) to ubiquinol (CoQ_10_H_2_), the ubiquinol directly reducing lipid peroxyl radicals to terminate lipid peroxidation; concurrently, FSP1 also participates in a noncanonical redox cycle of vitamin K and regenerating the oxidized α-tocopheryl radical (vitamin E) to its non-radical form to against ferroptosis [56, 143]. In addition, squalene and di/tetrahydrobiopterin (BH2/BH4) mediated inhibition of lipid peroxidation, may acting as endogenous radical-trapping antioxidants [56] (Fig. 1). The DHODH-CoQH_2_ pathway, localized to the outer surface of mitochondrial inner membrane, catalyzes the conversion of dihydroorotate (DHO) to orotate (OA) while simultaneously refreshing CoQH_2_ to clear lipid radicals [144].

### Targeting lipid peroxidation

According to above, therapies targeting lipid peroxidation primarily fall into two categories: radical-trapping antioxidants (RTAs) and inhibitors targeting enzymes such as LOXs and ACSL4.

### Radical-trapping antioxidants (RTAs)

Unlike traditional antioxidants, which stabilize free radicals primarily by donating electrons, RTAs physically capture and neutralize radicals by forming stable complexes with them [145]. Upon the conceptualization of ferroptosis, ferrostatin-1 (Fer-1), a RTA was discovered through a small molecules library screening [1] and its function is to scavenge the alkoxyl radicals [146]. Another RTA, liproxstatin-1, H-atom abstracted from liproxstatin-1 by radical occurs preferentially at the aromatic amine site (1’-NH) and liproxstatin-1 radical is easily regenerated to the active reduced form by endogenous ubiquinol, preventing secondary damage by free radicals [147]. Recent studies reveal that 7-dehydrocholesterol (7-DHC, a cholesterol precursor), as a RTA, effectively shields (hloride) lipids from autoxidation and subsequent fragmentation by using the conjugated diene, ultimately inhibiting ferroptosis, thereby presenting a novel metabolic target for therapeutic intervention [145, 148, 149].

### Targeting LOXs therapy

Recent advances have revealed a growing repertoire of molecules that critically regulate ferroptosis, including drug candidates from high-throughput screens, rationally designed enzyme-targeting compounds, and bioactive constituents extract from botanical, especially natural Chinese herbs.

LOXs, a class of non-heme iron-containing dioxygenases, mediate PL oxidation in ferroptosis. Humans express six LOX isoforms: ALOX5, ALOX12, ALOX12B, ALOX15, ALOX15B, and ALOXE3 [31]. Inhibitors of LOXs primarily function through three mechanistic classes: (1) directly integrated with LOXs; (2) modulation of post-translational modifications (e.g., acetylation, phosphorylation); (3) disruption of protein-protein interactions in LOX-containing complexes to destabilize functional assemblies (Table 2). ALOX5 promotes pancreatic cancer invasion and metastasis by driving tumor-associated macrophage M2 polarization via the JAK/STAT pathway, however, the ALOX5 inhibitor zileuton, currently the only approved agent in this class, can suppress these effects [150]; Bin Wang et al. reported that Nordy (dl-nordihydroguaiaretic acid compound), another ALOX5 inhibitor, induces differentiation and inhibits self-renewal in glioma stem-like cells [151]; Clau (Clausenamide) was found to inhibit lipid peroxidation and ferroptosis by blocking ALOX5 phosphorylation and nuclear translocation [152]. ALOX12 represents another therapeutic target, IMA-1 treats NASH by disrupting the interaction between ALOX12 and acetyl-CoA carboxylase 1 (ACC1) [153]; ML355, a targeted ALOX12 inhibitor, alleviates lung ischemia-reperfusion injury by reducing endothelial ferroptosis-mediated neutrophil extracellular trap (NET) formation [154]; separately, it impairs leukemia stem/progenitor cell function by disrupting nicotinamide adenine dinucleotide phosphate (NADPH) homeostasis, inducing oxidative stress and damage in CML HSPCs and committed cells by targeting ALOX12–12-HETE [155]; Baicalein protects against cisplatin-induced acute kidney injury (AKI) by inhibiting ALOX12-mediated ferroptosis [156]; hydrogen sulfide was proved to protect myoblasts from ferroptosis by inhibiting ALOX12 acetylation [157]; compound 99089, a potent and selective 12/15-LOX inhibitor, represents a promising candidate for developing anti-stroke therapeutics [158]. For ALOX15, there are many new development: Baicalein, a bioactive flavonoid from Banxia Xiexin Decoction, ameliorates CPT-11-induced gastrointestinal dysfunction by suppressing ALOX15-mediated ferroptosis [159]; Chicoric acid (CA), a natural ALOX15 inhibitor, suppresses ferroptosis in asthmatic by inhibiting ALOX15 expression [160]; Tianma Gouteng Granules (TG), a clinically prescription of traditional Chinese medicine, ameliorate behavioral deficits and dopaminergic neurodegeneration in Parkinson’s disease models by directly inhibiting ALOX15 activity and attenuating lipid peroxidation-driven ferroptosis [161]; Inhibition of ALOX15 by PD146176 suppresses epithelial-mesenchymal transition (EMT) in eosinophilic chronic rhinosinusitis with nasal polyps [162] and protects male germ cells against 4-hydroxynonenal (4-HNE)-induced protein damage [163]; Isochlorogenic acid C, a prominent component in Danzhi Xiaoyao Powder, mitigates macrophage phospholipid peroxidation in the stress-induced tumor microenvironment by inhibiting the ALOX15/PEBP1 complex [164]; FerroLOXINs are specifically designed inhibitors that selectively target the pro-ferroptotic 15LOX-PEBP1 complex, but not 15LOX alone [165]; Scutellarein alleviates chronic obstructive pulmonary disease (COPD) by inhibiting ferroptosis through iron chelation and ALOX15 interaction [166]; α-Tocopherol not only functions as a RTA but also regulates ALOX15: it inhibits ALOX15 activity by binding to 87^th^ Leu and suppresses ALOX15 expression [167].

**Table 2:** Summary of inhibitors target LOXs and ACSL4.

Therapeutic target	Drug name	Mechanism	Refs.
ALOX5	Zileuton	Regulate M2 polarization	[150]
	Nordy	Induces differentiation and inhibits self-renewal of glioma stem-like cells	[151]
	Clau	binding to ALOX5 Ser663 site and preventing the phosphorylation by PKCα	[152]
ALOX12	IMA-1	Interrupting the interaction between ALOX12 and ACC1	[153]
	ML355	Reducing endothelial ferroptosis-mediated NET formation; Disrupts NADPH homeostasis and induces oxidative stress and damage in CML HSPCs and committed cells	[154, 155]
	Baicalein	Inhibiting ALOX12-dependent ferroptosis	[156]
	H_2_S	Inhibiting ALOX12 acetylation	[157]
	Compound 99089	Ability to selectively target h12/15-LOX	[158]
ALOX15	Baicalein	Reduce lipid peroxidation and Fe^2+^ accumulation	[159]
	Chicoric acid	Suppresses ALOX15 expression	[160]
	Tianma Gouteng granules	May direct inhibition of ALOX15	[161]
	PD146176	Specific inhibition of ALOX15	[162, 163]
	Isochlorogenic acid C	Inhibit the ALOX15/PEBP1 complex	[164]
	FerroLOXINs	Act selectively on complex 15LOX/PEBP1	[165]
	Scutellarein	Chelating iron and interacting with ALOX15	[166]
	α-tocopheryl	Binds with 87th leucine of ALOX15 and inhibits ALOX15 expression	[167]
ACSL4	Baicalein	Upregulated the expression of GPX4 and ACSL3, suppressed the expression of ACSL4	[168]
	Rosiglitazone	Specific inhibitor	[58, 169, 170]
	Pioglitazone	ACSL4 inhibitor	[171]
	Troglitazone	ACSL4 inhibitor	[171]
	Sertaconazole	ACSL4 inhibitor	[172]
	PRGL493	Inhibits ACSL4 activity	[173]
	compound AS-252424	Directly binds to the glutamine 464 of ACSL4 to inhibit its enzymatic activity	[60]
	Berberine	Binds with ACSL4	[174]
	PKCβII	Phosphorylates ACSL4 at Thr328	[175]
	Fisetin	Inhibites ACSL4 transcription activity	[176]

### Targeting ACSL4 therapy

ACSL4, an enzyme that synthesizes PUFA-containing phospholipids, also regulates their production. Baicalein acts not only as a pan-LOX inhibitor but also modulates other enzymatic targets, including ACSL4 [168]; multiple studies confirm that rosiglitazone inhibits ferroptosis by suppressing ACSL4 [58, 169, 170]; like rosiglitazone, pioglitazone and troglitazone-both thiazolidinediones, act as pharmacological inhibitors of ACSL4 [171]; among FDA-approved compounds screened, sertaconazole emerged as a potent ACSL4 inhibitor [172]; PRGL493, an ACSL4 inhibitor developed through homology modeling and virtual screening, demonstrated potent inhibitory activity in validation studies [173]; using nanoparticle-based delivery, compound AS-252424 directly binds glutamine 464 of ACSL4 to inhibit its enzymatic activity, thereby suppressing lipid peroxidation and ferroptosis [60]; Berberine (BBR), an isoquinoline alkaloid historically used to treat diarrhea, has gained attention for its diverse pharmacological effects. Recently, it was found to inhibit ferroptosis in vascular endothelial cells by targeting ACSL4 [174]; post-translational modifications of ACSL4 critically regulate its activity, exemplified by PKCβII-mediated phosphorylation [175]; the natural flavonol fisetin ameliorates fibrotic kidney disease in mice by suppressing transcription activity of ACSL4 mediated-tubular ferroptosis [176].

### TfR mediated endocytosis therapy

Given TfR’s role in mediating Tf and ferritin endocytosis, making us to explore the probility of this mechanism as a potential therapeutic strategy. The primary therapeutic strategy involves enhancing drug uptake in target cells to achieve desired therapeutic outcomes. Sorafenib (SOR), a frontline hepatocellular carcinoma (HCC) therapeutic, induces ferroptosis through inhibition of solute carrier family 7 member 11 (SLC7A11). Nanovesicles engineered for surface Tf-Fe³⁺ display and SOR encapsulation (SOR@TF-Fe³⁺ NVs) achieved TfR-directed HCC targeting, enhancing tumoral iron/SOR accumulation and ferroptosis induction while maintaining favorable liver distribution [177]. Increasing intracellular iron levels to promote ferroptosis is another effective treatment. T10@Clav nanodrugs-engineered by conjugating Tf-targeting peptide T_10_ to cross-linked lipoic acid vesicles, hijack circulatory transferrin Tf for tumor-specific delivery. This system elevates labile Fe²⁺ via: (i) TfR-mediated iron import, and (ii) dihydrolipoic acid mediated Fe²⁺ regeneration from vesicle degradation, synergistically amplifying ferroptosis [178]. Another key therapeutic strategy involves enhancing intracellular accumulation of anticancer agents within malignant cells. Fluorinated 21 hloride[salophene]iron(III) complexes (salophene = N,N′-bis(salicylidene)-1,2-phenylenediamine) exhibit promising anticancer activity. Cellular uptake of the complexes appears to be mediated by TfR1. Critically, all complexes significantly decreased metabolic activity across tested cell lines derived from ovarian cancer, breast cancer, and leukemia [179]. RSL3 induces ferroptosis via GPX4 inhibition [139], while TfR1-mediates ferritin endocytosis promotes ferroptosis by increasing intracellular iron [137]. Consequently, the combination of RSL3 and ferritin therapy holds promise for enhanced therapeutic efficacy by simultaneously inhibiting the GPX4 antioxidant defense and elevating pro-ferroptotic iron levels. A bio-inspired protein nanocomplex was engineered by conjugating naturally occurring bovine serum albumin (BSA) with ferritin via acidity-responsive glutaraldehyde linkers. BSA provides multiple anchoring points for efficient RSL3 loading, while ferritin promoted TfR1-mediated cellular uptake. The nanocomplex significantly enhances antitumor efficacy without inducing observable adverse effects, highlighting its potential for clinical development of synergistic ferroptosis inducers [180].

### Ferritin related therapy

Combining different cell death modalities represents an effective strategy for treating diverse cancers. A novel carrier-free nanodrug, nanoparticle ferritin-bound erastin and rapamycin (NFER), was prepared via emulsion. In vitro studies demonstrated NFER’s robust ferroptosis-inducing capability through GPX4 downregulation and concomitant lipid peroxidation accumulation, and rapamycin-mediated autophagy within NFER acted synergistically to amplify ferroptosis [181]. Emerging evidence indicates that combining photothermal therapy (PTT) with ferroptosis induction represents a promising therapeutic strategy against drug-resistant tumors. In a recent study, triple-functional nanoparticle (I@P-ss-FRT) integrating thermal therapy, ferroptosis induction, and magnetic resonance imaging (MRI) capability was developed. Drug-resistant cancer cells exhibited significantly enhanced uptake of I@P-ss-FRT and synergistic sensitivity to PTT/ferroptosis co-activation. Notably, I@P-ss-FRT plus near-infrared (NIR) achieved optimal tumor suppression, accompanied by decline of GSH/GPX4 and accumulation of lipid peroxides at tumor sites in vivo [182]. Analogous to Tf, Ferritin-Hijacking Nanoparticles (Ce6-PEG-HKN15) was engineered by utilizing homing peptide, the nanoparticles spatiotemporally direct endogenous ferroptosis for synergistic anticancer therapy [183]. In addition, the traditional Chinese medicine dihydroartemisinin was revealed to trigger ferroptosis in cancer cells via autophagy-dependent ferritin degradation (ferritinophagy), liberating iron to amplify lipid peroxidation through Fenton reactions [184]. Sono-photodynamic therapy (SPDT) generates cytotoxic reactive oxygen species (ROS) under dual light/ultrasound activation, inducing multimodal cell death. This prompts us to consider the attempt of coupling SPDT and dihydroartemisinin therapy. Yilin Zheng et al. report a facile construction of ferritin-based nanosensitizer FCD through co-encapsulating chlorin e6 (Ce6) and dihydroartemisinin within horse spleen ferritin [185]. GPX4 and FSP1 constitute two dominant defense systems against ferroptosis, and dual inhibition of these two targets significantly enhances therapeutic efficacy against malignancies. A genetically engineered murine heavy-chain ferritin (mHFn) carrier mHFn@RSL3/Ifsp1 was designed to co-deliver RSL3 (GPX4 inhibitor) and iFSP1 (FSP1 suppressor) to tumors via TfR1(highly expressed on malignant cells)-mediated endocytosis. Simultaneous inhibition of both GPX4 and FSP1 antioxidant pathways overwhelms cellular redox homeostasis, triggering catastrophic lipid peroxidation cascades that culminate in synergistic ferroptosis [186].

### Traditional Chinese medicine (TCM) related therapy

**Fig. 5:**
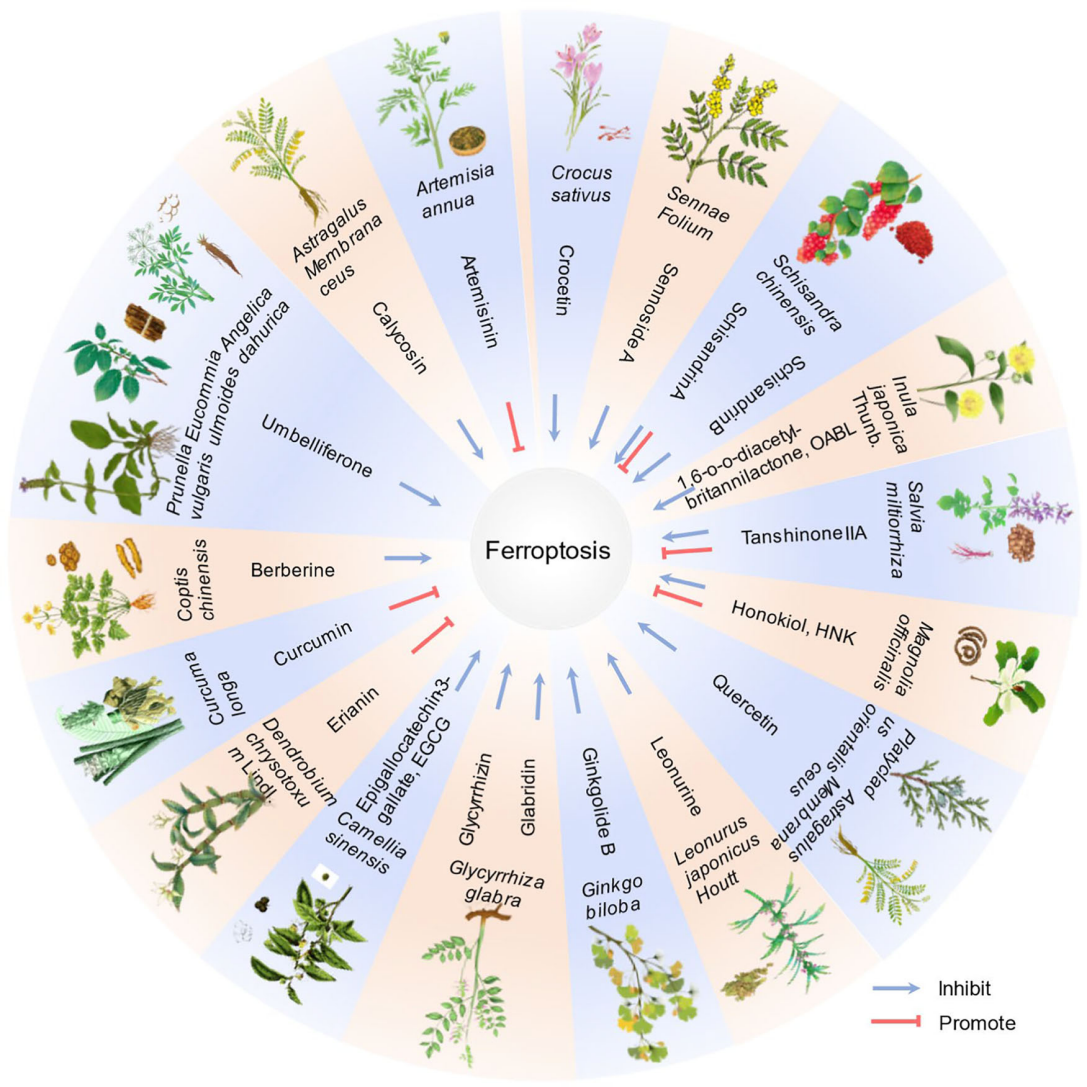
Pharmaceutical ingredients function of TCM in ferroptosis. This figure summarizes 19 traditional Chinese herbs and their associated bioactive compounds, with documented effects on ferroptosis regulation based on current research. Note: As research advances, additional bioactive compounds and regulatory functions in ferroptosis are anticipated to be identified. This diagram should be updated accordingly to reflect new evidence.

Complementing conventional treatments, research on natural TCM ingredients targeting ferroptosis has expanded significantly, yielding notable therapeutic effects (Fig. 5 and Table 3). Artemisinin and its derivatives, known as first-line antimalarials, also effectively target ferroptosis-related diseases due to their unique chemical structure [187]. Herbal ingredients exhibit divergent roles in ferroptosis regulation: inhibitors include artemisinin [187], calycosin [188], umbelliferone [189], berberine [190], leonurine [191] etc. while inducers comprise curcumin [192], erianin [193], etc. We further observed that the same herbal ingredient can exert divergent effects on ferroptosis via distinct signaling pathways. Honokiol (HNK), for instance, induces ferroptosis by suppressing GPX4 [194] or upregulating HMOX1 [195], while inhibiting ferroptosis through AMPK/SIRT1/PGC-1α pathway activation [196]; Tanshinone IIA, induces ferroptosis via p53-mediated SLC7A11 down-regulation [197] but inhibits ferroptosis through Nrf2 signaling pathway activation [198]; Schisandrin A, attenuates ferroptosis by AdipoR1/AMPK-ROS/mitochondrial damage [199] while activates ferroptosis via AMPK/mTOR pathway [200]. We further observed that single herbs contain multiple bioactive compounds, for example: astragalus membranaceus, yields both calycosin and quercetin; glycyrrhiza produces compound glycyrrhizin and glabridin; schisandra chinensis contains schisandrin A and schisandrin B (Table 3), consequently, this compositional complexity necessitates cautious in selecting herbal treatments. In addition, multiple studies confirm that nuclear factor erythroid 2-related factor 2 (Nrf2) signaling which regulates core ferroptosis components, including GPX4, system Xc⁻, FSP1 [201], mediates herbal ingredient effects on ferroptosis (Table 3), a mechanism further validated in traditional Chinese herbal therapy.

**Table 3:** Traditional Chinese herbal ingredients in the treatment of ferroptosis.

Chinese herb name	Pharmaceutical ingredient	Structural formula	Mechanism
*Artemisia annua*	Artemisinin C_15_H_22_O_5_		Degrade intracellular ferritin to upregulate free iron level; upregulate p53 to inhibit system Xc^-^ function [187]
*Astragalus Membranaceus*	Calycosin C_16_H_12_O_5_		Suppress ACSL4 [188]; alleviates ferroptosis by activating the Nrf2/SLC7A11/GPX4 signaling [265];
*Angelica dahurica*, *Eucommia ulmoides, Prunella vulgaris*	Umbelliferone C_9_H_6_O_3_		Inhibiting ferroptosis through activation of the Nrf-2/HO-1 pathway [189]
*Coptis chinensis*	Berberine C_20_H_18_NO_4_^+^		Inhibiting ferroptosis by up-regulating NRF2/SLC7A11/GPX4 [266] and targeting ACSL4 [174]; BBR suppressed ferroptosis via promoting GPX4 expression [190]
*Curcuma longa*	Curcumin C_21_H_20_O_6_		Induces ferroptosis via activating autophagy [192]
*Dendrobium chrysotoxum* Lindl.	Erianin C_18_H_22_O_5_		Exerts anticancer effects by inducing Ca^2+^/CaM-dependent ferroptosis [193]; inducing ferroptosis via NRF2 inactivation [267] and blocking the JAK2/STAT3/SLC7A11 signaling pathway [268]
*Camellia sinensis*	Epigallocatechin-3-gallate, EGCG C_22_H_18_O_11_		Inhibiting ferroptosis by increasing NRF2 and GPX4 expression [269]
*Glycyrrhiza glabra*	Compound Glycyrrhizin C_42_H_62_O_16_		Inhibiting ferroptosis via the HMGB1/GPX4 Pathway [270], HMGB1-TLR4-GPX4 [271] and Keap1/Nrf2/HO-1 Pathway [272]
	Glabridin C_20_H_20_O_4_		Repressed ferroptosis by increasing SOD and GSH activity, and GPX4, SLC7A11, and SLC3A2 expression [273]
*Ginkgo biloba*	Ginkgolide B C_20_H_24_O_10_		Alleviates ferroptosis by inhibiting GPX4 ubiquitination [274]; inhibiting ferroptosis by disrupting NCOA4-FTH1 interaction [275]
*Leonurus japonicus* Houtt	Leonurine C_14_H_21_N_3_O_5_		Alleviating ferroptosis by activating the Nrf2 pathway [191]; reduces ferroptosis by increasing GPX4 and Nrf2 expression [276]
*Platycladus orientalis, Astragalus Membranaceus*	Quercetin C_21_H_20_O_11_		inhibits ferroptosis by inhibiting the expression of ATF3 [277] and Nrf2 [278]; inhibits ferroptosis by downregulating phosphorylation of PI3K, AKT, mTOR [279]
*Magnolia officinalis*	Honokiol, HNK C_18_H_18_O_2_		induces ferroptosis by reducing the activity of GPX4 [194] or upregulating HMOX1 [195]; inhibits ferroptosis by activating AMPK/SIRT1/PGC-1α pathway [196]
*Salvia miltiorrhiza*	Tanshinone IIA C_19_H_18_O_3_		induces ferroptosis through p53-mediated SLC7A11 down-regulation [197]; inhibits ferroptosis through activating Nrf2 signaling pathway [198]
*Inula japonica* Thunb.	1,6-o-o-diacetyl-britannilactone, OABL C_19_H_26_O_6_		inhibits ferroptosis by increasing the GSH level [280]
*Schisandra chinensis*	Schisandrin A C_24_H_32_O_6_		Attenuates ferroptosis by AdipoR1/AMPK-ROS/mitochondrial damage [199]; activating ferroptosis by AMPK/mTOR pathway [200]
	Schisandrin B C_23_H_28_O_6_		Attenuates ferroptosis via AMPK/PGC1α/Nrf2 signaling pathway [281]; inhibits ferroptosis through SIRT1/p53/SLC7A11 signaling pathway [282]; reduce ferroptosis by inhibiting oxidative stress [283]
*Sennae Folium*	Sennoside A C_42_H_38_O_20_		restrains TRAF6 level to modulate ferroptosis [284]
*Crocus sativus*	Crocetin C_20_H_24_O_4_		Alleviates ferroptosis by facilitating Nrf2 nuclear translocation [285]; moderating ferroptosis via Nrf2/GPX4 pathway [286]

In short, ongoing phytochemical and mechanistic studies of TCM will progressively uncover: (1) undiscovered therapeutic compounds, (2) sophisticated action pathways, and (3) expanded clinical applications. Last, expanded ferroptosis research will uncover potent therapeutic strategies, particularly for cancer; however, their clinical application necessitates overcoming persistent translational barriers.

## Conclusion

Collectively, all these above significantly advance our understanding of ferroptosis. The peroxidation of glycerophospholipids, generating reactive peroxyl radicals and cytotoxic protein adducts, plays a decisive role in ferroptosis. Accumulating evidence demonstrates that targeting ferroptosis holds significant therapeutic promise, particularly for treating cancers and other ferroptosis-related diseases.

Ferroptosis is a complex event involving multiple cellular processes, such as glycerophospholipids metabolism, redox homeostasis, autophagy, signal transduction, etc. while redox homeostasis regulation is the key to mastering ferroptosis. Therapeutic induction of ferroptosis via promotion of lipid peroxidation is primarily employed against various cancers. In contrast, inhibition of lipid peroxidation to suppress ferroptosis provides a therapeutic avenue for conditions including Parkinson’s disease, fibrosis, fatty liver, and ischemia-reperfusion injury. Key molecular targets for promoting lipid peroxidation include GPX4, FSP1, SLC7A11, Tf/TfR, and LPCAT1 etc.; conversely, targets for inhibiting lipid peroxidation encompass pro-ferroptotic enzymes ALOXs, ACSL4, or administering RTAs, and glutathione precursors (e.g., NAC/D-NAC) etc.

Notably, certain pharmacological agents exhibit context-dependent bidirectional effects on ferroptosis. For instance, TCM compounds including Honokiol, Tanshinone IIA, and Schisandrin A demonstrate both pro-ferroptotic and anti-ferroptotic activities through distinct molecular mechanisms. This functional duality underscores the inherent complexity of TCM-based ferroptosis modulation, necessitating integrated evaluation of disease pathology, drug mechanism of action, and clinical outcomes for therapeutic optimization. Furthermore, nanoparticle-based drug delivery systems for ferroptosis modulation are emerging as a rapidly advancing therapeutic strategy. Concurrently, novel ferroptosis-related molecular targets continue to be identified [202].

In conclusion, targeted modulation of ferroptosis represents a promising therapeutic paradigm for precision disease treatment.
